# Supplementary material for: Foraging through multiple nest holes: An impediment to collective decision-making in ants
Source: PLoS One. 2020 Jul 1;15(7):e0234526. doi: 10.1371/journal.pone.0234526 (PMC7329192; doi:10.1371/journal.pone.0234526)
Supplement: S1 Table — The table lists the main findings of the current paper (Two feeders) and of a previous paper by Lehue et al 2020 (One feeder) that used an identical experimental setup but different ant colonies. A positive or a negative sign means that the foraging characteristics is respectively favored or hampered by the opening of a second nest entrance. A sign put between brackets means that only a trend (not statistically significant) was observed. 0 means that no impact was found. NA: Not available data due to the lack of well-defined trail over the foraging area (Two feeders) or the lack of opportunity of food choice (One feeder). (DOCX) [file pone.0234526.s001.docx]

|  | One Feeder (1M) | Two feeders (1M Vs 0.1M) |
| --- | --- | --- |
|  | One -> Two entrances | One -> Two entrances |
| Total Outflow | **0** | **+** |
| Trail emergence and duration | **(-)** | **NA** |
| % ants reaching food | **(-)** | **-** |
| % ants at the best food source | **NA** | **-** |
| Retrieved food amount | **(-)** | **-** |
| Sugar yield per forager | **(-)** | **-** |

Supplementary table 1: Impact of multiple nest entrances on ants’ foraging towards either a single feeder (1M sucrose solution) or two feeders of different quality (1M Vs 0.1M sucrose solution) . The table lists the main findings of the current paper (Two feeders) and of a previous paper by Lehue et al 2020 (One feeder) that used an identical experimental setup but different ant colonies. A positive or a negative sign means that the foraging characteristics is respectively favored or hampered by the opening of a second nest entrance. A sign put between brackets means that only a trend (not statistically significant) was observed. 0 means that no impact was found. NA: Not available data due to the lack of well-defined trail over the foraging area (Two feeders) or the lack of opportunity of food choice (One feeder).
