# Supplementary material for: Foraging through multiple nest holes: An impediment to collective decision-making in ants
Source: PLoS One. 2020 Jul 1;15(7):e0234526. doi: 10.1371/journal.pone.0234526 (PMC7329192; doi:10.1371/journal.pone.0234526)
Supplement: Attachment — Submitted filename: Responses to reviewers final.docx [file pone.0234526.s002.docx]

Reviewer #1: In this manuscript, Lehue and Detrain continue their exploration of the role of the nest entrance on ant foraging. Specifically, they ask how having one or two nest entrances affects the recruitment and foraging dynamics of Myrmica rubra colonies foraging on two sucrose feeders of different qualities (0.1 vs 1M. They report that while having two nest entrances causes a larger surge of recruitment to newly discovered food sources, foraging efficiency suffers for multiple reasons. Key amongst them is that foraging is less well-focussed on the high quality feeder. In addition, fewer recruits successfully find the food source within 10 minutes of leaving the nest.

I found this work interesting, well carried out, and generally well written. The methodologies used were appropriate, and I could detect no critical methodological issues. Almost all of the conclusions drawn are well supported by the data (for one exception, see below). In all, this is a good contribution in an understudied area, and I recommend publication with moderate revisions. However, some key literature is not discussed, and there are methodological choices which may limit the generalisability of the results. Moreover, one of the main results (increased recruitment in 2-entrance nests) did not replicate in the authors previous paper, and the explanation for this was not fully convincing. I also have a series of more minor points about the figures, the supplement, and some grammatical suggestions. I hope the extensive comments I have made show how much I value this work.

**We are very grateful to the reviewer for his positivity and insightful comments. We hope that the changes we have made to our manuscript satisfactorily address these concerns. To clarify the take-home message, we reworded the title of the MS.**

Introduction
The introduction is rather long and generic, with the whole first page and a half being basically suitable to any collective decision-making paper on ants. One example is lines 51-56. The authors might consider cutting this generic aspect of the introduction down. They will need this for the next point:

**We acknowledge that the introduction was a bit too long. So, as suggested by referee 1, we shortened it by removing lines 51-56 (in the original MS) and we reedited lines 455-459 in the discussion (in the revised MS). Nevertheless, we would like to keep a short generic framework to introduce our study as it makes sense for the wide readership of PlosOne.**

Two papers are key to understanding this work. The first is a previous recent paper by the authors (Lehue et al. 2020), and the second is Pinter-Wollman (2015). The previous work needs to be discussed in much more detail – specifically, all of the main results of that experiment need to be listed, and it should be mentioned that the experiments were identical except for the food sources used. Indeed, the discussion might even benefit from a small table listing the main findings of the current paper and this previous paper, so that we can ‘see’ this body of work in one place.

**Referee 1 is right when telling that the current MS is a continuation of a recently published paper (Lehue et al 2020). Actually, the experimental setup is identical in many aspects (same nest design, same foraging area,….). However, it is also important to say that the ant colonies used in these two studies were not the same. Thus, one cannot readily compare the absolute values (e.g. outflow values) obtained in the present studies with those obtained in Lehue et al 2020. Furthermore, the aim of these two studies was not the same. In Lehue et al 2020, our main objective was to assess the impact of multiple entrance on the emergence of a collective structure (the trail) outside the nest. In the current study, we investigate how multiple entrances influence the ability of a colony to make optimal choices. Keeping in mind that one cannot directly compare the absolute values obtained in the two papers, we nevertheless followed referee 1’s advice by adding a a supplementary table that lists the main findings from Lehue et al 2020 and current MS. This table helps to visualize the impact of a second nest entrance when either a single food source (Lehue et al 2020) or a choice between two food source was offered to ant colonies(current MS). Under the light of our previous work, we discuss the main findings of current MS such as the impact on the outflow of foragers at lines 421-427, the ability to reach food at lines 424-426 and the retrieved food amount at lines 443-455.**

I was surprised that the Pinter-Wollman paper was not mentioned at all, considering its deep parallels to the current work. Specifically, the paper shows that as connectivity increases, so does recruitment speed. This is very similar to what is reported in the current paper. This paper should thus be discussed in some detail both in the introduction and in the discussion.

**Pinter-Wollman paper is actually a pioneering study about the impact of nest structure on collective behavior. Her work however differs from the current study as for how the nest structure is taken into consideration. Pinter-Wollman work demonstrates that the nest topology- in particular the connectivity of the entrance chamber- can increase the dynamics of recruitment. The current paper is focused on the structure of physical interface between nest interior and the outside environment. In our nest design (unlike Pinter-Wollman study), we wanted to set apart the potential influence of connectedness by using nests with single large chamber. That being said, we agree with referee 1 that reference to Pinter-Wollman was missing and added it at lines 417-419 and 499 in the discussion.**

Ln 33 – information sources. **Done. Thanks.**

Ln 33-34 – some of these references are not about social insect decision making, but the sentence is only about that. **Many thanks for noticing. We changed the sentence accordingly (line 30)**

Ln 91 – kept, not hosted.

**We made the change at line 85 as well as in other sentences in the main text and in the caption of table 1. Thanks for the suggestion.**

Methods
The food sources were rather close to each other. The resultant pheromone trails from the multiple nest entrances thus cross over quite extensively. I wonder, if the angle between the food sources and the nest were larger (at the extreme, if the food sources were at each side of the nest), whether the effects described in this paper would be present at all. This is probably an issue worth raising in the discussion – how generalisable are these results?

**This is a very interesting question. Most likely, a larger angle between the food sources will reduce crossing between trails originating from multiple entrances. At the extreme, one may expect that a collective choice will no longer take place with one of the two entries leading to one of the two food sources. Such a result is expected insofar as the recruitment trails originating from the two entrances will be spatially well-distinct and therefore the likelihood for recruits to “compare” their relative attractiveness will be close to zero. In the current study, increasing the angle between feeders would not be appropriate since we wanted to investigate how the nest interface may influence the accuracy of collective choices made by the whole colony. To make a choice possible, we had to design an experimental setup that enabled ant foragers to compare the information sources (recruiting stimuli inside the nest and chemical trails outside the nest) for the two available food sources. This required to place food sources relatively close to each other. We added a sentence on this issue (Lines 479-484) in the revised MS**

A second methodological concern is the size of the nest: these are quite small nests (300 workers, 8cm diameter), and the distance between the two entrances is quite small (c. 3cm, right?). Thus, as the author notes, the ‘activation area’ of the two nest entrances overlap, possibly leading to increased recruitment. However, in a natural nest, which covers more ground and is composed of a tunnel system, multiple nest entrances likely do not overlap in effect. Again, it is important to ask: how realistic and generalisable are the results, given these methods? I would argue that, if the nest were composed of a tunnel system, and the two nest entrances were in two somewhat separate cavities (as likely occurs in nature), very different dynamics might occur. This does not mean that the current experiment is meaningless – far from it – but it does suggest that we don’t have the whole story here (see the Pinter-Wollman paper, for example). I think this issue needs to be raised explicitly. Maybe in the future, this experiment could be repeated with a nest composed of tunnels and cavities…

**The reviewer is correct that different results can be expected in the case of a tunnel system and more remote entrance chambers behaving as separate cavities, each being involved in their own recruitment process and with little (or no) overlapping between trails. Please see Lines 479-484 dealing with this issue**

**As for the location of food sources, the distance between the two entrances was quite small in order to test how such a slight change of the nest interface may neverthess alter the accuracy of collective decision-making. As for the realism of this nest configuration, we made several observations on natural nests of M.rubra (this field study is currently submitted for publication) and we found that the number of active entrances per colony ranged from a single one up to six holes, with some nest entrances being aggregated into clusters. Inside a cluster, the average distance between entrances was 5cm, which is quite similar to the distance used in the current study. This information has been added to the MS at line 92-98 and 114-116.**

Ln 198 – define “ants present at a food source” more formally. Touching the food source? Within 1cm of the food source?

**Thanks for the remark. This information is now provided at lines 132-135 and 151-152.**

Results
Table 1 – while later in the manuscript it is claimed that the amount of sucrose taken in by colonies is given (ln 370-373), this does not seem to be what is reported here. I note, for example, that 26.1 + 0.8 = 26.9 (column 3). This suggests that the 0.1M sucrose has not, in fact, been reduced by a factor of 10 to account for having only 10% as much sucrose as 1M. The key foraging metric here, after all, is not weight of sucrose solution returned, but the weight of sucrose returned. 1 molar sucrose has ten times as much sugar as 0.1M. Please amend this, and rerun the analysis, if necessary. It will only strengthen the results.

**Thanks for this comment. Table 1 shows both the weight of solution ingested (regardless of its molarity; second data row) and the weight of sucrose ingested (third data row). This last measure takes into account the difference in molarity between the feeders meaning that the amount of 0.1M solution is actually reduced by a factor of 10 compared to 1M solution in order to estimate the amount of sucrose “units” that were retrieved. For instance, for the one-entrance nest, the ingested weight of 1M solution was 85mg thus resulting in a weight of sucrose of 342 (1M) X 0.085= 29.07 g. As for the amount of ingested solution at the 0.1M feeder (27mg), the corresponding amount of ingested sucrose was of 34.2 (0.1M) X 0.027= 0.92 g. When revising table 1, we found an error in the molecular weight of sucrose retained for the calculation. Following referee 1 comment, we fully revised table 1.**

A large concern for me was that one of the main results – the increase in forager mobilisation from 1 to 2 nest entrances – was not replicated in Lehue et al. (2020) (discussed in ln 409-411). This does not make much sense to me, and the hypothesis proposed by the authors is unconvincing. They argue, if understood them correctly, that more food sources would mean a higher rate of returning workers, so higher recruitment and activation. Firstly, given the cross-shaped feeders and presumably no queuing at the feeders, this doesn’t sound likely. Secondly, it is not clear to me why have one or two nest entrances should affect the total number of returning foragers, regardless of the number of food sources. One possibility is that, with only one food source or only one entrance, recruitment is downregulated, due more encounters with ants on the path (Czaczkes et al. 2013) and at the food source (Wendt et al. 2020). However, luckily the authors have all the data needed to support or reject their hypothesis in their videos. If they were to count the number of returning foragers (assessable by abdomen distention) within the first 10 minutes, their hypothesis predicts many more returning foragers in the two-entrance configuration. Don’t forget to collect this data blind to treatment, though, as abdomen distention is quite hard to assess in M. rubra! As an aside, examining the raw data from this and the previous paper, I note that that the foraging dynamics of the colonies are very similar for one food source one entrance (previous paper), one food source two entrances (previous paper), and two food sources two entrances (this paper), with the two food sources two entrances standing out, with double the recruitment. This suggests there is something special about this configuration – it might be worth presenting the data from the 2020 paper again here for comparison.

**Unfortunately, for practical reasons of limited magnification and definition of images, video-recordings do not allow to see the distension of foragers’ abdomen and therefore we cannot confidently count the returning foragers.** **As regards our first hypothesis to account for the doubling of recruitment in Two-entrances/Two feeders situation, we regret that our explanation was unconvincing for the two referees. Therefore, we removed the whole § about the “leaky integrator” hypothesis and we fully reedited lines 412 to 427.**

**We agree with referee 1 that differences in foragers’ flows between the current study and Lehue et al 2020 could be due to the fact that, with only one food source or only one entrance, recruitment is downregulated by more encounters with ants on the path (Czaczkes et al. 2013) or at the food source (Wendt et al. 2020). This possibility and the suggested references have been added to the MS at line 421-423.**

**Concerning the foraging dynamics to a single source (previous paper) and to two food sources (current MS), one should keep in mind that these experiments were carried out on different colonies thus preventing a direct comparison of absolute numbers of ants mobilized. However, one can compare the impact of nest configuration (one VS two nest entrances), separately in each of these two conditions of food availability (as reported in the new supplementary table).**

**As highlighted by referee 1, the 2 source-2 entrance was the only configuration leading to a doubling of ant mobilization. In our opinion, this is most probably due to small differences in recruitment rates at the early steps of foraging, such differences being self-amplified over time. A slightly higher recruitment rate to two feeders may be due to the spacing of resources over a wider area than a single source what 1°) increases the likelihood for ants to discover food and to recruit nestmates and 2°) what reduces encounters of returning foragers on a common path and hence their downregulating effect on the recruitment ( cf referee comment). Furthermore, two entrances increase the total number of ants that can be potentially recruited. Taken all together, the 2 sources-two entries condition is the one that most favors ants’ mobilization. Accordingly, the whole § was fully reedited from lines 412 to 427; a supplementary table was created and the suggested references were added.**

Ln 468 – replace “dispatching of multiple information” with “distributing incoming information” /Ln 498 – replace “to draw” with “for creating”

**Thanks. These changes were done.**

Supplement – data. I applaud the authors for providing the raw data. All papers should do this. However, the data is presented in a very hard to use format in the supplement. Firstly, all labels are in French – this should be amended. Secondly, the data has not been entered in a ‘tidy’ manner – tidy having a specific definition in data entry (https://vita.had.co.nz/papers/tidy-data.html). In short – every column should be a variable, and every row an observation. Please also provide metadata (information about what each column is). This all may sound terrible and pedantic (I apologise!), but it really is very important, and will facilitate data analysis and visualisation for you in the future.

**New files of raw data are provided on Zenodo. The revised data table does not fit to all the recommendations provided in the suggested website but rows and columns are now labelled in English with a more extensive title.**

Figure 2, 3, 4A&B– please add mean connect lines. Indeed, this data is not normally distributed, so you should probably be using medians and quartiles, not means and SD.

**We chose to use means and SD since it improves the clarity of data trends (further confirmed by non-parametric tests) . All the figures were re-edited by adding lines connecting mean values.**

Figure 4 A and B – These figures make seeing the main comparison (1 vs 2 nest entrances) very difficult, and are more appropriate for exploring the dynamics, which is a side issue here. Perhaps just present the total mean time for one and two nest entrance? Indeed, just mean proportion ants at feeder (two bars) captures everything we need to know, but a figure similar to figure 8 would also be ok.

**We think that the dynamics of ant presence at the two feeders deserve to be kept in the MS as they provide information about the global level of food exploitation. Furthermore, these figures provide a visual support to the finding that a two-entrance nest reduces the efficiency of ants to reach the food source. Indeed, even though ants’ mobilization doubled in two-entrance nests, the total numbers of foragers at the feeders was similar to the one observed in one-entrance nest. For these reasons, we would prefer to keep figure 4A and 4B in the MS.**

Figure 4C – why does this now have connection lines but no error ribbons?

**It’s an oversight. Thanks for noticing.**

REFERENCES CITED.

Czaczkes TJ, Grüter C, Ratnieks FLW (2013) Negative feedback in ants: crowding results in less trail pheromone deposition. J R Soc Interface 10:. https://doi.org/10.1098/rsif.2012.1009

Lehue M, Collignon B, Detrain C Multiple nest entrances alter foraging and information transfer in ants. Royal Society Open Science 7:191330. https://doi.org/10.1098/rsos.191330

Pinter-Wollman N (2015) Nest architecture shapes the collective behaviour of harvester ants. Biology Letters 11:20150695. https://doi.org/10.1098/rsbl.2015.0695

Wendt S, Kleinhölting N, Czaczkes TJ (2020) Negative feedback: Ants choose unoccupied over occupied food sources and lay more pheromone to them. Journal of The Royal Society Interface. https://doi.org/10.1098/rsif.2019.0661

**We thank the reviewer for his insightful comments which have helped to substantially improve our manuscript.**

____________________________________________________________

Reviewer #2: I found this to be a carefully conducted study. It shows that ants are less capable of choosing a richer food source over a weaker one, when the ants have more than one exit hole through which they can leave their nest. Two related things concerned me about the study, which may well be answerable.

**We thank the reviewer for this positive comment.**

1. The short time period during which it seems that the colonies were examined. Do ants improve if they are allowed to forage freely for a few days with two entrances before being tested? You say they were allowed ad lib food for a time with two entrances but without specifying the details. Would be nice to find out if ants do improve over time, by, for instance, coming to use just one of the exits.

**Each colony was first kept in the tested nest configuration for four resting days by being allowed to forage freely and to feed *ad libitum. T*hereafter, the same colony was starved for 48 h and ultimately, its foraging response was quantified in this nest configuration. We did not record the flows at the two entrances during the 4-days resting period but ants did not come to use just one exit. We clarify the experimental procedure at lines 141-145.**

2. Could the lack of adaptation to the problems that you set in part be a consequence of abnormal conditions in the artificial nests during testing? E.g. they haven't time to sort themselves out?

**A resting period of four days should be enough time for an ant colony to reorganize itself as a response to a new nest configuration. Indeed, in a previous paper (Lehue & Detrain 2019, reference 32), we found that just after the opening of new entrances, the incoming and outgoing ant flows spread betwen all the open entrances but some entrances were temporarily favored. However, after only 20 hours, foragers dynamically reorganized themselves and the flows of ants become similar between all the open entrances. Following referee 2 comment, this information is given at lines 141-143. We hope that this alleviates the reviewer’s concerns.**

Line by line commentary.

Line 78. Somewhere around here you need a bit of natural history about nest entrances. There are obvious examples of nests with one entrance (e.g. Cataglyphis fortis). Are there any with several exit holes (Formica rufa ?) and in such cases do individual ants always use the same hole to enter or exit by same hole or do they have other methods of successful recruitment?

**We now provide some information about entrances of *M.rubra* nests at lines 92-94 based on some observations that we made in the field. We found that several exit holes can be used by the same colony. As regards the spatial fidelity of foragers to the same hole, we do not have such information for the current lab experiments. In the field, we observed a spatial fidelity to feeder/ nest hole but for longer time scale, for longer distances between feeders and for a more individual-based foraging than in the current study. For these reasons, these field data (under review in a submitted MS) on spatial fidelity are not directly transposable here.**

Line 100. Be nice to know rough numbers of ants in wild colonies to compare with the 300 in your experimental ones.

***M. rubra* nests composed of 100 up to 1,500 workers (based on our personal observations and Elmes 1973). For the nests that were dug under stones or under wood logs, the superficial nest chambers housed a few hundred individuals and consisted of a large single chamber or of multiple chambers, separated by loose walls or well- defined ridges (personal observations). This information has been added at lines 94-98 as well as reference to Elmes work.**

Line 118. Do you have evidence that 48 hours is long enough for ants to be acclimated? Might they organise themselves to cope with 2 entrances if given a longer period?

**As above mentioned, we found in a previous study that ants reorganized themselves already 20h after the opening of new nest holes (Lehue & Detrain 2019, reference 32). Please also see lines 140-142**

Line 140. Did ants have the chance to explore the arena during this ad lib period or was the food adjacent to the nest and exploration prevented by a barrier? Relevant to general comment at the start of these comments.

**Yes, the ants were allowed to freely explore the whole arena during four days before the experiments. There was no barrier that prevented the exploration. This information is provided at lines143-144.**

Line 240. Was there any correlation between asymmetry of entrance use and proportion of visits to the high concentration feeder?

**Referee 2 raises a very interesting point. In the two-entrance configuration, ant flows ranged from an almost perfectly symmetrical use of the two entrances to an asymmetrical use of a preferred entrance (lines 246-249). Noticeably, we found that a higher asymmetry of entrance use favoured the selection of the best food source. This can be seen on figure 7 and is reported at lines 341-346.**

Line 272. Do you have proportions of how many ants in the one and two nest conditions returned after feeding and how many returned empty stomached?

**This point has also been addressed by referee 1. Unfortunately, we do not have such data and our video-recordings did not allow us to see whether ants were empty stomached or had filled their crop. This would deserve to be done in future studies.**

Line 282 Fig. 4 would be helpful if you label in legend and fig the three conditions (legend) and arrows (fig) as 'a', 'b' or 'c'.

**We added the labels. Thanks for the suggestion.**

Line 291. I'm curious whether you videoed the foraging and if so whether you can say anything about trail use in the two entrance conditions.

**In a previous study (Lehue et a 2020) with a single food source, we found that two-entrances altered the trail building process, especially when foragers equally used the two passageways. In the current study, using two feeders made the spatial distribution of trails even more fuzzy with no clear-cut trail structure that emerged. This is now specified at lines 238-239.**

Line 293 'as the 0.1' not 'than the 0.1'

**Done. Thanks.**

Line 299 Again question about asymmetry of entrance use and proportion of ants reaching 1M sucrose. P = 0.053 doesn't mean no preference but just a weak preference.

**Following referee 2 comment, the sentence was reworded at lines 305-309.**

Line 305. Don't like 'repartiton' prefer something like 'how ants distributed themselves between..'

**We made the change. Thanks for the advice.**

Line 333. So there is a correlation between asymmetry of entrance use and food preference. Be helpful to a reader to mention it briefly earlier - around line 240.

**Referee 2 is correct that there is a clear-cut correlation. Following referee 2 advice, we tried to mention it around line 240 but it makes the sentence rather confusing since results on the relative exploitation of food sources are reported later in the MS (around line 313). Therefore, we prefer to keep the first version and let the reader “discover” the correlation at line 341 .**

Line 348. suggest: 'drank about twice as much from the 1M than from the .1M feeder'

**Thanks. At line 357, we did not use exactly the suggested sentence since we compared the probability for ants to drink and not the ingested food amount.**

Line 394.409 'twice as many nestmates'

**We made the change. Thanks.**

Line 419 -425. I'm uncertain about the argument made here. You need more detail to be convincing that in this experiment the outflow of foragers depends highly on encounters between returning ants and potential foragers. Are there sufficient returning foragers to generate the timing of maximum outflow in Fig 2 when there are two exits? And do returning ants return to their exit hole or are they indiscriminate? Have you compared outflow from one and two hole nests when holes are opened after a period of being shut and there is no food to find. Does the total peak then differ between the two nests? Para needs a conclusion of what the answer might be.

**A similar point was raised by referee 1. Unfortunately, it is impossible to see on video-recordings, the distension of the abdomen and therefore we cannot confidently count the returning foragers. As regards the timing of the experiment, we are confident that it was sufficient to generate the maximum outflow of ants, even when there were two nest exits. Indeed, the current experiments were carried out over a period (two hours) that usually encompasses the whole process of recruitment in *M.rubra* ant species. One can also see on figure 2 that the recruitment of additional workers decreased, already 20 minutes after the start of the experiment. This means that the peak outflows were triggered during the first half-hour of foraging. Unfortunately, here we got no information about the fidelity of foragers to a nest hole since we did not follow returning ants during their successive trips to the nest. Finally, concerning the outflows of exploring ants when there is no food to find, we found in a previous study (Lehue & Detrain 2019, reference 32), that the cumulated outflow (not the peak value) increased but in less than proportional way when changing from a one-hole nest to a three-holes nests. Based on comments of both referees, this section was fully reedited at lines 412-427.**

Line 430 'at the same time'

**Done. Thanks for the suggestion.**

**We are grateful to the reviewer for his/her positive suggestions. We hope that the points raised have all been satisfactorily addressed.**
